# Research on the reliability of the overall structure of submerged radial steel gate

**DOI:** 10.1371/journal.pone.0322452

**Published:** 2025-06-26

**Authors:** Yanchun Yu, Ziyan Zhao, Zhonghao Zhang, Weidong Chen, Yi Zhong, Yongxin An

**Affiliations:** 1 School of Water Conservancy & Civil Engineering, Northeast Agricultural University, Harbin, China,; 2 School of Astronautics and Architectural Engineering, Harbin Engineering University, Harbin, China; Southwest Petroleum University, CHINA

## Abstract

The safety assessment of steel gate structures holds significant social importance and economic advantages. The safety of steel gate structure is influenced by numerous random factors, so it is necessary to carry out the reliability analysis of steel gate structure. Currently, research on the reliability of steel gate structures predominantly focuses on individual components, cross-sections, or localized areas, and there are relatively few studies on the reliability of the overall structure of the steel gate. Furthermore, most of the studies only consider the role of hydrostatic pressure load, often underestimating the impact of other dynamic load combinations. Therefore, this study calculates the strength and stiffness of the overall submerged radial steel gate structure under various loading conditions, including hydrostatic pressure, hydrodynamic pressure and sediment pressure. Additionally, it conducts the analysis of the reliability regarding the overall structure of the submerged radial steel gate. The results indicate that the maximum stress and displacement values conform to the allowable limits established by relevant design codes, with the maximum stress occurring at the support hinge. The reliability analysis of the submerged radial steel gate structure was performed by considering different load combinations and material parameters as random variables, and the structure was safe at a 95% confidence level. Additionally, the sensitivity analysis of the steel gate structure was conducted, and the results showed that the hydrodynamic pressure and elastic modulus had a higher degree of influence on the results of displacement calculations, while other parameters had a relatively low degree of influence. Similarly, the hydrodynamic pressure had a greater influence on the results of stress calculations, while other parameters had a relatively small influence.

## Introduction

There are numerous large and medium-sized hydraulic structures in China, some of which incorporate radial steel gates. These gates have either reached or are approaching the end of their useful life. The reliability of these gates remains uncertain, with some posing significant hidden risks. Nonetheless, they continue to be utilized for practical and financial reasons. Consequently, the safety assessment of these gates cannot be postponed, as it holds substantial social significance and economic benefits.

Domestic and foreign scholars have extensively studied the reliability of steel gate structures [1–7] and achieved a large number of research results. Foreign scholars Mlakar and Bryant (1990) have made significant contributions, emphasizing that corrosion leads to a reduction in reliability. Padula (1994), Kathir (1996), Binder (2001), Patev (1998), etc. based on the corrosion-resistant nature of steel, established the nonlinear damage model of steel in their respective point of views and assessed the reliability of steel gates. In terms of time-varying reliability analysis of gates, Patev et al. analyzed the time-varying reliability of steel gates by using time-varying reliability analysis method and hazard function method.

In the calculation method, foreign scholars have developed their own understandings and breakthroughs. Freudenthal’s (1946, 1956, 1966) research on reliability theory began with his article “The Safety of Structures”, in which he highlighted a new direction for structural design in 1966: designing structures based on probability theory. In 1969, Cornell introduced the second-order moment method, which primarily describes the formula for the reliability index β, serving as a metric for measuring structural safety. This marked the transition of structural reliability theory into engineering practice. In the same year, Rackwitz and Riessler from West Germany proposed the equivalent normalization method (JC method), designed to address the challenges posed by non-normally distributed random variables in reliability calculations. Subsequently, the response surface method was introduced, with Box proposing a technique to replace the original limit state surface of the structure. In the field of engineering structural reliability, significant contributions have been made by Wong (1985), Faravelli (1989), and Bucher and Bourgund (1990) towards refining the response surface method. Leveraging advancements in computer technology, Isukapalli (1998) integrated this method with stochastic techniques, resulting in the development of the stochastic response surface method.

Scholars Zhou Jianfang and Li Dianqing [8] evaluated the dynamic reliability of steel gates using a hierarchical analysis method that accounts for the effects of time variation on gate reliability. Xia Nianling derived a life prediction formula for steel components based on the permissible stress design method. Ren Yushan and Mou Xinhe investigated the remaining life of steel gates through the lens of a time-varying reliability index. Additionally, Li Dianqing and Tang Wenyong [9] predicted the lifespan of structural members in in-service steel gates, proposing a prediction method that considers the randomness of load and resistance. Furthermore, ongoing research by scholars both domestically and internationally has led to the establishment of robust calculation methods for structural reliability.

In the past five years, research on the reliability of hydraulic structures has yielded significant contributions. Zhu Yanzhi analyzed the fuzzy reliability of the ferry systems using the primary second-order matrix method. Li Ying [10] examined the reliability of seawall structures based on Monte Carlo method. Chen Quanhao [11] proposed further analysis and direction for the specific practice of structural reliability in the design of hydraulic structures. Liu Ming [12] assessed the reliability of rocky slope stability utilizing the random response surface method. Sun Bo [13] calculated the time-varying reliability of the planar steel gate system structure by optimizing the gate structure and assessing the gate’s reliability in conjunction with the reliability of the main beam, incorporating a non-linear corrosion law. Ping Yuan [14] studied the dynamic analysis of the process of stream ice impacting the arc gate and the comparison of the response of the arc gate under different impacting conditions. Chen Yang [15] studied the self-vibration characteristics and the effect of water flow pulsation on the plane steel gate under fluid-solid coupling. Chen Lin [16] investigated the mechanisms underlying the failure of the hydro-dynamic gate, proposing prevention and control measures to address these failures. The continuous failure process of the gate structure was analyzed, clarifying the failure mechanisms involved. Pei Duofei [17] using numerical analysis has analyzed the dynamic characteristics of the gate, the buckling analysis of the gate and the shock vibration response. Seonghyun Lim et al [18] introduced the (β-π) analysis method, which comprehensively evaluates the reliability and redundancy of individual structures. Chulyoung Kang et al [19] investigated the correlation between structural failures based on the demand parameter (EDP) in structural engineering. Bruce Ellingwood et al [20] provided a summary of structural reliability theory, highlighting its developmental applications and its critical role in guiding the structural engineering profession in addressing key aspects of practice.

Currently, design methods based on structural reliability theory play a significant role in various industries, including water conservancy, harbor management, and construction [21–26]. However, domestic research on the reliability of steel gates remains limited, existing research focus on the reliability of individual components, specific certain cross-sections, or localized areas, with relatively little attention given to the reliability of the overall structural system of steel gates. While the reliability analysis of the main beam system has established a foundation for understanding the overall structural reliability, it does not adequately represent the reliability of the entire system. To accurately assess the reliability of the overall structure of steel gates, it is essential to consider the influence of other components on the structural system. Therefore, in the study of the reliability of the overall structural system of steel gates, it is necessary to comprehensively consider the study of the various components of the steel gate structure in order to obtain the correct results of the study.

As the steel gate is composed of multiple components, making the analysis of its overall structural system reliability significant for research. Currently, there is limited research on the reliability of the overall structure of steel gates, and this aspect of the analysis and calculation in this area is relatively complex. Presently, several shortcomings exist in the reliability studies of steel gates. Common methods used in engineering practice include the first order reliability method (FORM), response surface method, Monte Carlo method, higher-order moment method, and neural network method. Further research and discussion are necessary to effectively apply structural reliability theory in the design of hydraulic steel gates. This paper will utilize the Monte Carlo method to conduct preliminary reliability research on the overall structure of submerged radial steel gates, thereby providing a theoretical foundation for the structural reliability analysis of hydraulic steel gates and demonstrating significant practical value.

## Static finite element analysis of submerged radial steel gate

### Finite element model of submerged radial steel gate

**Fig 1:**
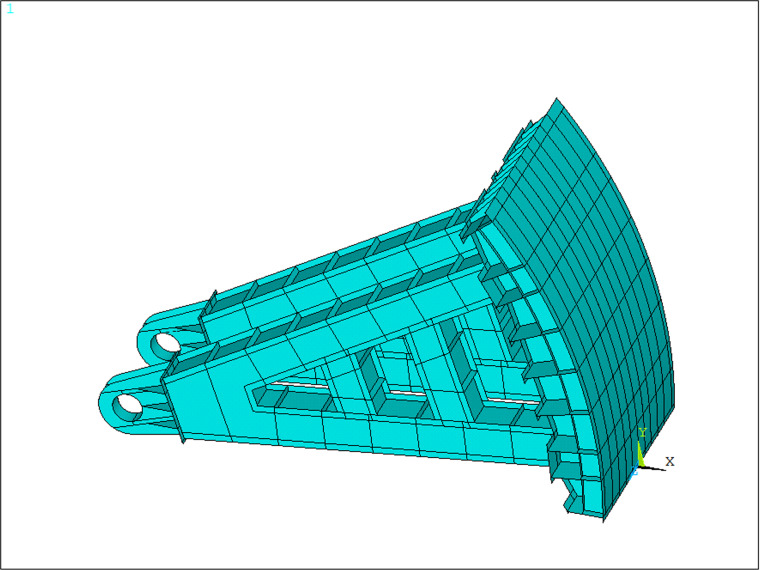
Model of submerged radial steel gate.

The steel gate model studied in this study is a submerged radial steel gate, which is based on the submerged radial steel gate prototype of the reconstruction project of a hydropower station. It features an aperture width of 6.58 m, a clear height of 9.92 m, a branch-hinge support span of 3.50 m, and a radius of the outer edge of the gate panel of 15.00 m. The steel thickness varies across the components of the gate: the panel has a thickness of 40 mm, The steel thickness varies across the components of the gate: the panel has a thickness of 40 mm, the beam steel thickness of 36 mm, and a branch-hinge steel thickness of 24 mm, and a branch-arm steel thickness of 50 mm. A structural finite element model of the submerged radial steel gate has been established based on the finite element software, as illustrated in Fig 1. The parameters pertaining to the material of the steel gate are detailed in Table 1.

**Table 1:** Material parameters of steel gate.

Steel grades	modulus of elasticity	Poisson’s ratio	density
Q235B	2.1 × 105 MPa	0.3	7850 kg/m^3^

**Fig 2:**
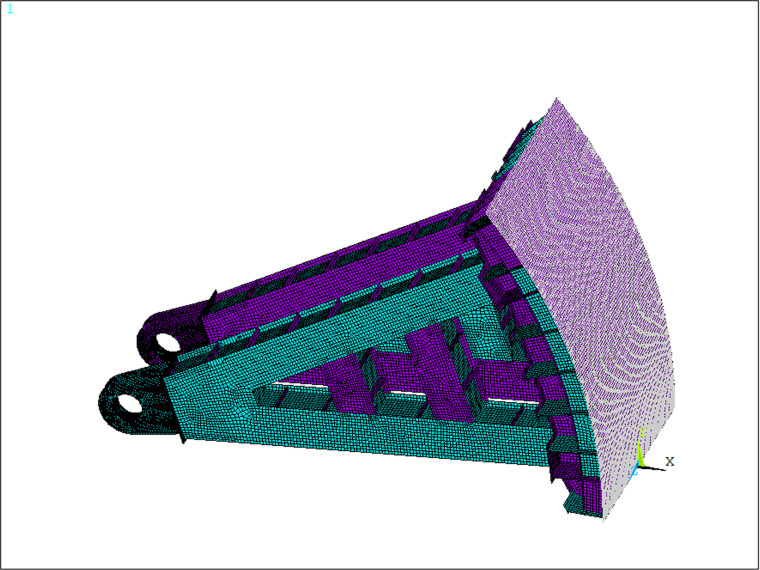
Mesh element of model of steel gate.

In the finite element model, the SHELL181 four-node shell element is employed, with a total of 100,205 elements and 97,468 nodes, and the mesh elements are illustrated in Fig 2.

**Fig 3:**
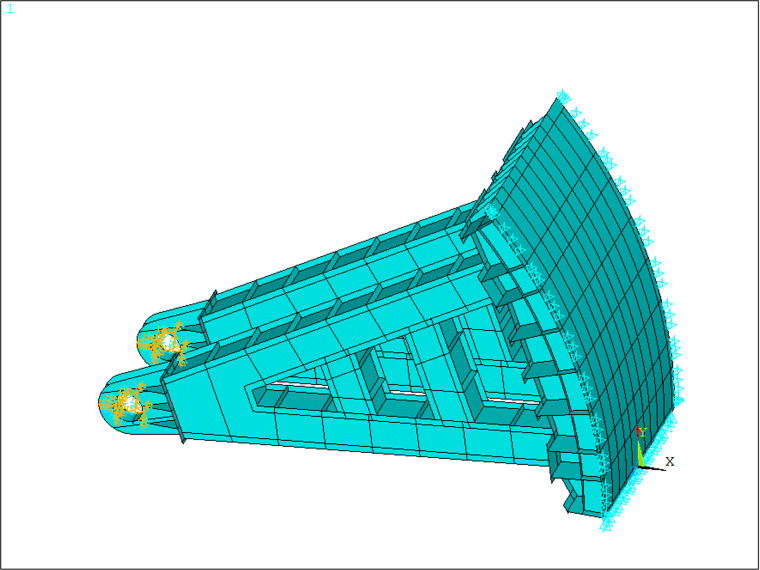
Schematic diagram of constraint conditions of submerged radial steel gate.

Considering the water blocking case of the gate, Y-direction constraints are applied at the bottom of the gate model, Z-direction constraints are implemented on both sides of the gate model, and fixed-end constraints are established at the supporting hinges, as illustrated in Fig 3.

### Load analysis of submerged radial steel gate

According to the Design specification for steel gates of Water and hydropower projects (SL74-2019) [27], In the design of gate structure, the loads acting on the submerged radial steel gate is mainly hydrostatic pressure. In order to make the analysis more comprehensive, the load combination of hydrodynamic pressure and sediment force is considered in the analysis. In this study, we consider a typical working state, which includes self-weight, hydrostatic pressure, hydrodynamic pressure, and sediment pressure.

According to relevant norms and literature [28,29], the load combination also considers the load effect ratio ρ. ρ is the ratio of the remaining load to the standard value of the hydrostatic pressure. Therefore, hydrostatic pressure is represented as variable *W*, hydrodynamic pressure is represented as variable *D*, sediment pressure is represented as variable *N*, and dead load is represented as variable *G*. The load combination and its corresponding ρ are shown in Table 2 [29].

**Table 2:** Load combination and ρ-value.

Load condition	Variate	Type of gate	ρ
Dead load + hydrostatic pressure + hydrodynamic pressure	*G* + *W* + *D*	open-roofed gate	*D*/*W* = 0.2
		Submarine gate	*D*/*W* = 0.1
Dead load + hydrostatic pressure + hydrodynamic pressure + sediment pressure	*G* + *W* + *D* + *N*	Submarine gate	*D*/*W* = 0.1 *N*/*W* = 0.2

### Strength and stiffness calculation of submerged radial steel gate

In order to ensure the safe commissioning of the gate structure, the strength of the gate is categorized according to the steel size grouping, outlined in the “Design specification for steel gates of water and hydropower” (SL74-2019) [27], In this calculation example, the thickness of each component, such as the the panel, falls within the second group of the steel size classification. For steel gates used in large-scale projects, taking into account the higher degree of safety, the allowable stress should be multiplied by an adjustment factor of 0.9 to 0.95, and the adjustment factor is taken as 0.95 in this calculation example.

In the strength calculation, the expression for the limit state design of the load carrying capacity is as follows:


$$\gamma_{0}\psi S_{d}\left(\gamma_{G}\cdot G_{k},\gamma_{Q}\cdot Q_{k},\alpha_{k}\right)\leq \frac{1}{\gamma_{dn}}R\left(\frac{f_{k}}{\gamma_{m}},\alpha_{k}\right)$$ (1)

In the formula, $\gamma_{0}$ is the importance coefficient of the structure, which is generally taken as 1.0; ψ is the coefficient of the design condition, which can be taken as 1.0 for the design condition of this study; S(·) is the function of the effect of the combination of roles; R(·) is the function of the structural design value of the resistance; $\gamma_{G}$ and $\gamma_{Q}$ represent the coefficients of the permanent action and the variable action. The partial coefficient of action reflects the adverse variation of action to its standard value, which is specifically expressed as the ratio of the design value of action to the standard value of action [30]. The coefficients of the common loads of the gates are shown in Table 3 [29]; $G_{k}$ and$Q_{k}$ represent the standard value of the permanent action and the variable action; $\alpha_{k}$ is the standard value of the geometrical parameters; $\gamma_{m}$ is the itemized coefficient of material properties; $\gamma_{m}=\frac{f_{k}}{f_{d}}$, the value is 1.036 for Q235 steel. $\gamma_{dn}$ is the structural coefficient for the corresponding nth combination of roles, which is 1.50 in this study.

**Table 3:** Partial coefficient of load [29].

Load type	Hydrostatic pressure	hydrodynamic pressure	wave pressure	sediment pressure	earthquake pressure
Partial coefficient	1.0	1.1	1.2	1.2	1.0

In the stiffness calculation, the limit state formula should be used, taking into account the design values of the load combinations. The resistance values are calculated as follows:


$$\frac{1}{\gamma_{dn}}R\left(\frac{f_{k}}{\gamma_{m}},\alpha_{k}\right)=\frac{235}{1.5\times 1.036}\approx 150\mathrm{MPa}$$ (2)

When examining the stiffness of a structure or member, the formula should be based on the normal service limit state design, while its results should be based on deformations, cracks, etc. The specific expression for normal use limit state design [30]:


$$\gamma_{0}S\left(G_{k},Q_{k},f_{k},\alpha_{k}\right)\leq C$$ (3)

In the formula, S(·) is effect design value function of the standard combination; *C* is functional limits for normal use of the structure or structural member.

The loading conditions and the corresponding standard and design values of loads for the steel gate model in this study are detailed in Table 4:

**Table 4:** Load Condition, Standard Value of Load and Design Value of Load.

Load condition	Standard value of Load	Load design value
Self-weight + hydrostatic pressure + hydrodynamic pressure + sediment pressure	Self-weight + hydrostatic pressure + 0.1*hydrostatic pressure + 0.2*hydrostatic pressure	1.2*Self weight + hydrostatic pressure + 0.1*hydrostatic pressure*1.1 + 0.2*hydrostatic pressure*1.2

According to the Unified Standard for Reliability Design of Structures of hydraulic engineering structures (GB 50199-2013) [30], the design expression for the limit state of normal use must be employed when calculating structural stiffness, and the standard load combination should be utilized to compute the combination value. For the submerged steel gate, the ratio of the maximum deflection to the calculated span of the gates and main beams must not exceed 1/750. In this model, the calculated span of the beam is 6.320 m, and the maximum allowable deflection is 8.4 mm.

**Fig 4:**
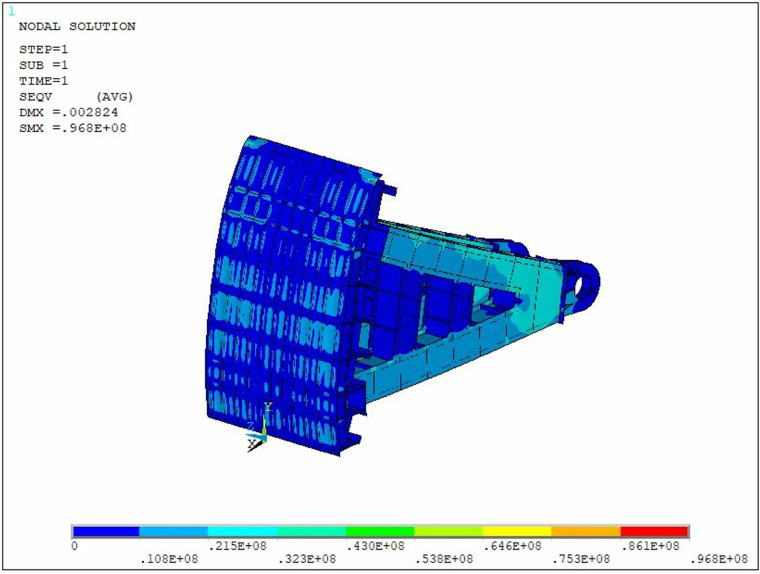
Stress nephogram of steel gate.

**Fig 5:**
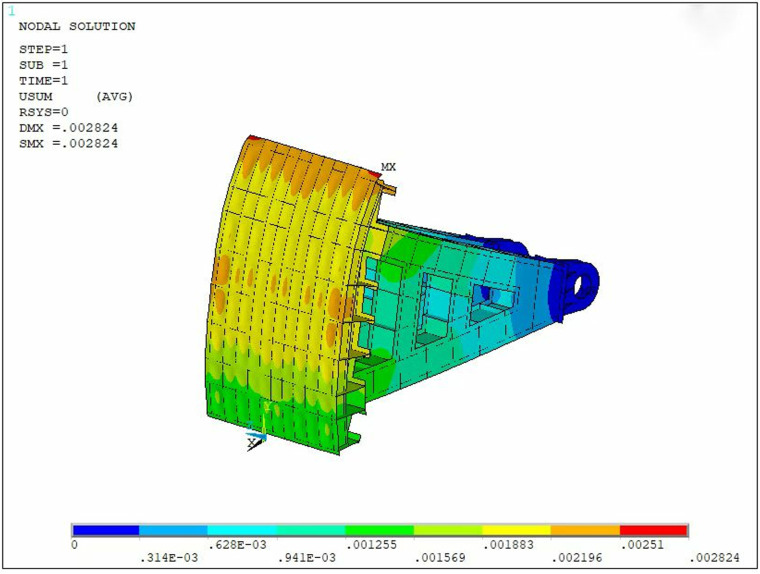
Displacement nephogram of steel gate.

The stress and deformation of the steel gate under the specified loading conditions were analyzed, and the stress and displacement cloud diagrams of the steel gate are illustrated in Figs 4 and 5.

The results of the above calculation indicate that the maximum stress in the gate structure under the above loading conditions is 97MPa, and the maximum stress occurs in the supporting hinge area, where stress concentration is likely to arise. Additionally, the two edges of the gate panel are identified as the locations of maximum displacement, with the structure experiencing a maximum displacement of 2.824 mm. According to the Design specification for steel gates of water and hydropower projects [27], the stresses and displacements of the steel gates meet the requirements for checking stiffness and strength.

## Reliability analysis of submerged radial steel gate

### The limiting state equation of submerged radial steel gate

The prerequisite for conducting a reliability analysis of submerged radial steel gates is to establish their limit state equations. According to the stress-strength interference theory, the limit state equations for submerged radial steel gates can be described as follows [31]:


$$Z=g(x_{1},x_{2},\cdots x_{n})=\left[\sigma \right]-\left[\sigma_{max}\right]$$ (4)

In the formula, $x_{1},x_{2},\cdots x_{n}$ are random variables, [σ] is permissible stress of materials of gates, $\left[\sigma_{max}\right]$ is maximum equivalent stress of gate. The three cases of limit state equation are as follows:


$$\left\{ \begin{array}{cc} Z>0 & Safestate\\ Z=0 & Limitingstate\\ Z<0 & Failurestate \end{array}\right.$$ (5)

To analyze the reliability of submerged radial steel gates, it is necessary to study the characteristics of the distribution of random variables that affect the reliability of the gate.

### Statistical parameters of load and material constant

Before conducting the reliability analysis of submerged radial steel gates, it is essential to first statistically analyze the common loads and material constants associated with these steel gates. The statistical parameters of the loads refer to the type of load distribution and allocation parameters. According to the relevant literature, the statistical parameters of gate loads [30] are specifically detailed in Table 5, and the statistical parameters of material constants are detailed in Table 6.

**Table 5:** Statistical Parameters of the Load.

Load type	notation	gate type	Average/standard value	coefficient of variation	distribution type
Hydrostatic pressure	W	Submarine gate	1.16	0.04	normal distribution
		open-roofed gate	1.08	0.085	normal distribution
Hydrodynamic pressure	D	Submarine gate	0.90	0.135	normal distribution
		open-roofed gate	0.55	0.45	normal distribution
sediment pressure	N	Submarine gate	0.49	0.30	lognormal distribution

**Table 6:** Statistical parameters of material constants.

Variate	average value	variable coefficient	Distribution type
modulus of elasticity	2.1e11	0.02	normal distribution
Poisson’s ratio	0.3	0.02	normal distribution
density	7850	0.02	normal distribution

Based on the statistics of load and material constants for the submerged radial steel gates, the reliability index of the structure is verified through Monte Carlo reliability analysis calculations. The limit state equations employed in the reliability calculation should be considered by the structural design to ensure the accuracy of the calculation results. The limit state equations are as follows:


$$R-S_{w}-S_{q}=0$$ (6)

In the table, $S_{w}$ is the load effect caused by hydrostatic pressure, and $S_{q}$ is the load effect caused by other loads (e.g., hydrodynamic pressure, sediment pressure, etc.).

Pressure loading is applied to the curved surface of the steel gate, with the hydrostatic pressure corresponding to a water depth of 30 meters. It is assumed that under this loading condition, the maximum stress inside the submerged radial steel gate structure will lead to structural damage if it exceeds the material’s yield limit. The damage criterion is as follows:


$$\sigma_{\max}\leq \sigma_{\text{s}}$$ (7)

In the formula, $\sigma_{\max}$ is the maximum equivalent force inside the steel gate structure and $\sigma_{s}$ is the yield strength of the material. The limit state function is:


$$Z\left(x\right)=\sigma_{s}-\sigma_{\max}$$ (8)

The failure state is defined as Z≤0. The Monte Carlo method was used for 10,000 simulated sampling calculations.

**Fig 6:**
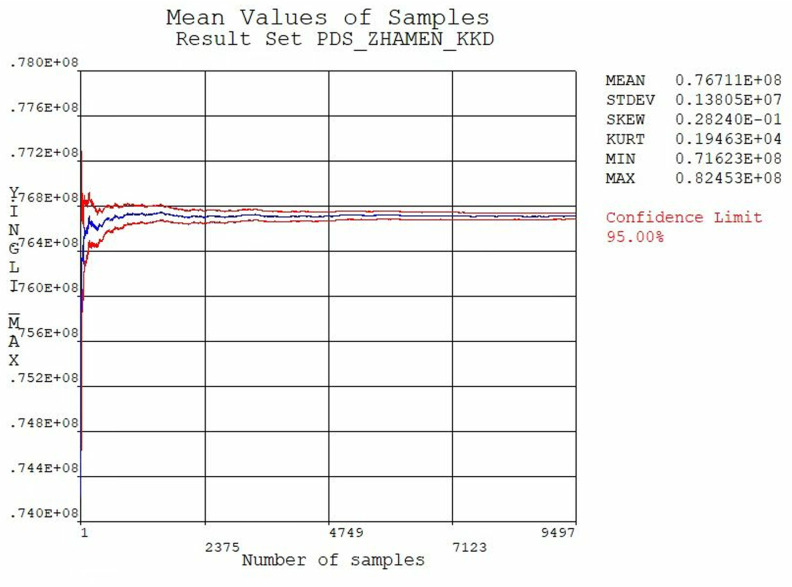
Average value of equivalent stress.

**Fig 7:**
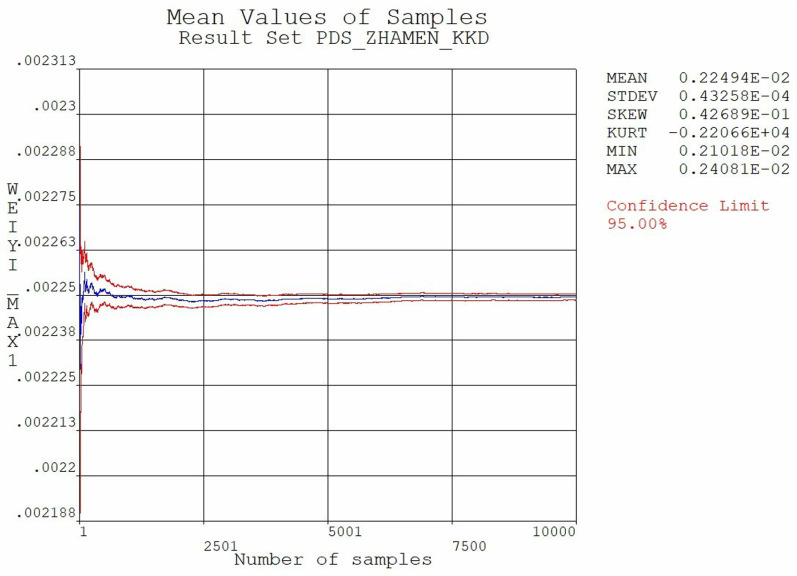
Average value of displacement.

The results are illustrated in Figs 6 and 7, In Fig 6, the horizontal axis represents the number of samples taken, while the vertical axis indicates the maximum stress value. Similarly, in Fig 7, the horizontal axis denotes the number of samples taken, and the vertical axis represents the maximum displacement value. By analyzing the stresses and displacements of the extracted samples under the condition, it is indicated that the gate is safe under the combined loading condition of self-weight, hydrodynamic pressure, hydrostatic pressure and sediment pressure. Furthermore, the calculation results reveal that the mean values of equivalent stresses and displacements gradually stabilize, marking that the number of sampling iterations is sufficient.

### Sensitivity analysis of submerged radial steel gate

**Fig 8:**
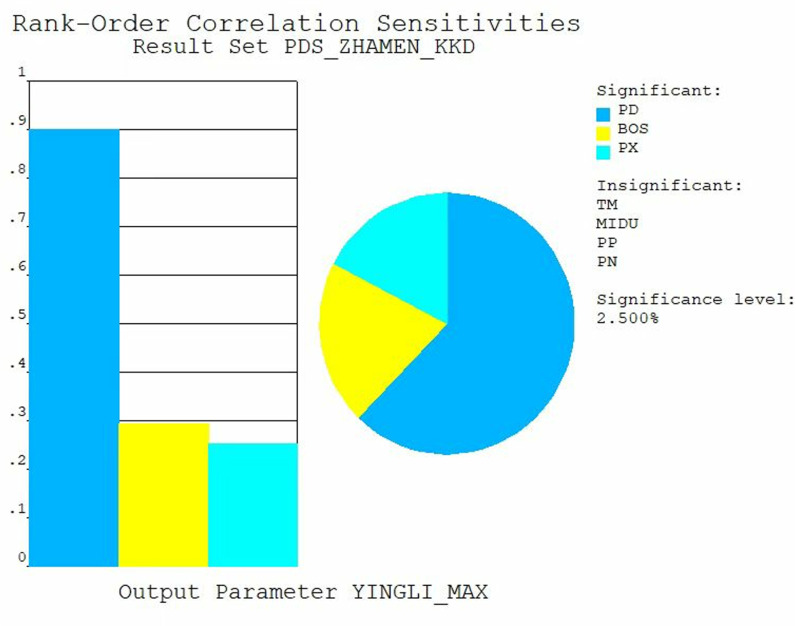
Sensitivity analysis of stress.

**Fig 9:**
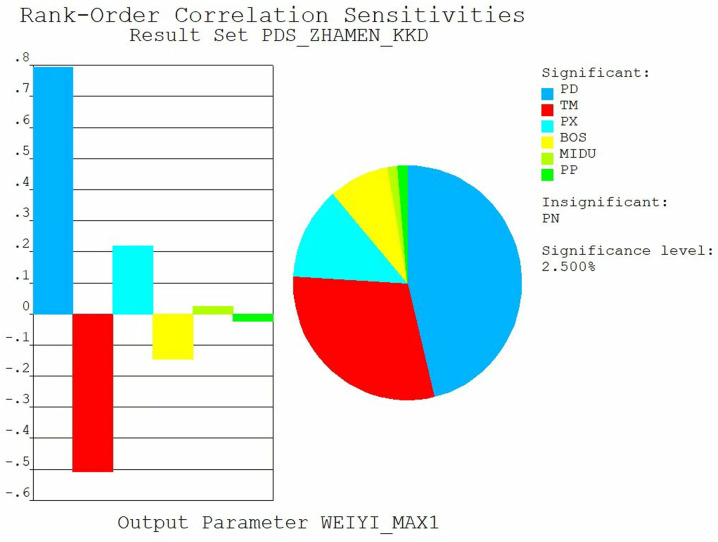
Sensitivity analysis of displacement.

Sensitivity analysis is a key step in analyzing the reliability of steel gates. Sensitivity analysis can reveal the response of steel gates in the face of different loads and working conditions, as well as identify the key factors affecting the performance of the steel gates. Generally, input parameters that exert an influence of less than 2.5% on output parameters are classified as having minimal relative influence, while those exceeding 2.5% are deemed to have significant influence. The sensitivity analysis of the output variables equivalent force and displacement illustrated Figs 8 and 9. In this study, PD denotes hydrostatic pressure, BOS represents Poisson’s ratio, PX signifies sediment pressure, TM indicates modulus of elasticity, MIDU refers to density, and PP stands for hydrodynamic pressure. The results indicate that the hydrodynamic pressure and elastic modulus significantly affect the displacement calculations, whereas the influence of other parameters is comparatively minor. Additionally, the hydrodynamic pressure plays a substantial role in the stress calculation results, while the impact of other parameters remains relatively small. Consequently, it can be preliminarily analyzed and concluded that hydrodynamic pressure is a critical factor affecting the reliability of gates.

**Fig 10:**
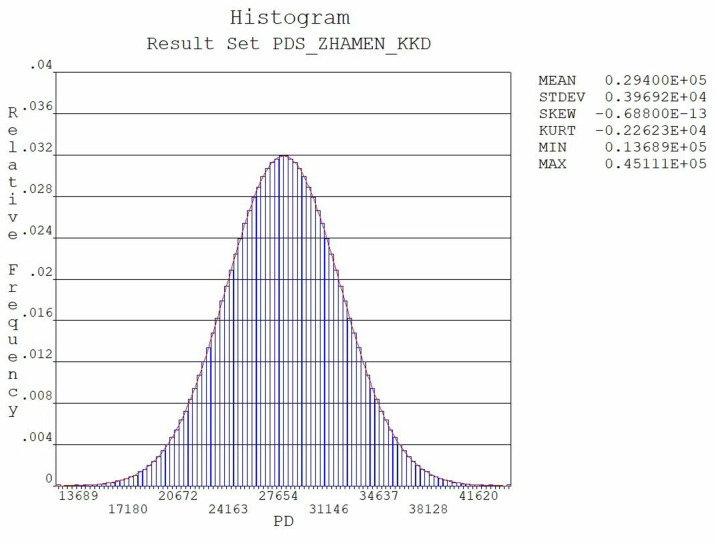
The column diagram of the distribution of hydrodynamic pressure.

The results of the sensitivity analysis indicate that hydrodynamic pressure is a significant factor affecting the reliability of the gates. Consequently, a histogram of the hydrodynamic pressure was plotted, as illustrated in Fig 10. The data distribution presented in the histogram is closely related to the normal distribution function curve, with no apparent breaks or jumps between the two. This observation suggests that the random errors in the data collection and processing are minimal, thereby indicating a high level of data reliability.

## Conclusions

This paper investigates the strength and stiffness analysis, as well as the structural reliability analysis of submerged radial steel gate under various loading conditions. The following conclusions were drawn:

1. The strength of the submerged radial steel gate structure was evaluated under various loading conditions. The results indicate that the maximum stress experienced by the steel gate structure is 97 MPa, which complies with the strength requirements outlined in the current Design specification for steel gates of water and hydropower projects [27]. Notably, the maximum stress occurs in the support-hinge section, which is susceptible to stress concentration phenomena. This observation suggests that the support-hinge area of the steel gate may be the first component to sustain damage, as stress concentration is likely to manifest in this region.
2. The strength of the submerged radial steel gate structure was evaluated under various loading conditions. The results indicated that the maximum displacement of the steel gate structure was 2.824 mm, which complies with the stiffness requirements outlined in the current Design specification for steel gates of water and hydropower projects [27].
3. A reliability analysis of the submerged radial steel gate structure was conducted, taking into account hydrostatic pressure, hydrodynamic pressure, and sediment pressure as random variables in the calculations. Additionally, material parameters such as modulus of elasticity, Poisson’s ratio, and density were also treated as random variables. The results indicate that the steel gate structure is safe under these working conditions. Furthermore, a sensitivity analysis of the steel gate structure was performed, revealing that the hydrodynamic pressure and elastic modulus significantly affect the displacement calculations, whereas the influence of other parameters is comparatively minor. Additionally, the hydrodynamic pressure plays a substantial role in the stress calculation results, while the impact of other parameters remains relatively small.

**Table:** Notation

G	self-weight (N/m)
W	hydrostatic pressure (N/m)
D	hydrodynamic pressure (N/m)
N	sediment pressure (N/m)
p	ratio of other loads to hydrostatic pressure (-)
$\gamma_{0}$	importance coefficient of the structure (-)
ψ	design condition coefficient (-)
S(·)	combined effect function (-)
R(·)	design value function for structural resistance (-)
$\gamma_{G}$	partial coefficients of permanent action (-)
$\gamma_{Q}$	the partial coefficients of variable action (-)
$G_{k}$	standard values of permanent action (-)
$Q_{k}$	standard values of variable action (-)
$\alpha_{k}$	standard value of geometric parameters (-)
$\gamma_{m}$	partial coefficient of material properties (-)
*C*	functional limits value of serviceability limit states for a structure or structural member (-)
[σ]	permissible stress of materials of gates (Pa)
$S_{w}$	load effect caused by hydrostatic pressure (-)
$S_{q}$	load effects caused by other loads (-)
$\sigma_{\max}$	maximum equivalent stress of the steel gate (Pa)
$\sigma_{s}$	yield strength of materials (Pa)

## Supporting information

S1 File The command flow of reliability analysis of submerged radial steel gate. (PDF)
