# Supplementary material for: Research on the reliability of the overall structure of submerged radial steel gate
Source: PLoS One. 2025 Jun 26;20(6):e0322452. doi: 10.1371/journal.pone.0322452 (PMC12200700; doi:10.1371/journal.pone.0322452)
Supplement: S1 File — (PDF) [file pone.0322452.s001.pdf]

The command flow of reliability analysis of submerged radial steel gate

```
finish
/clear

*creat,zhamen_kkd,pds

RESUME,'gate','db','.'

tm=2.1e11
bos=0.3
midu=7850

pp=1000*9.8*30
px=9810
pd=1000*9.8*30*0.1*1.1
pn=1000*9.8*30*0.2*1.2

/prep7
mpdele,ex,1
mpdele,prxy,1
mpdele,nuxy,1
mpdele,dens,1

mp,ex,1,tm
mp,prxy,1,bos
mp,dens,1,midu

/solu
!LSCLEAR,ALL
allsel
antype,0
acel,,9.8*1.2,
cmsel,s,mianban
nsla,s,1
sfgrad,pres,,y,30,-px
sf,all,pres,pp
sf,all,pres,pn
sf,all,pres,pd
alls

!cmsel,s,a_jiao
```

```
!nsla,s,1
!d,all,all
!allsel
!nsel,s,loc,x,0
!nsel,r,loc,y,0
!d,all,uy
!allsel

!cmsel,s,l_bian
!nsll,s,1
!d,all,uz
!allsel

solve

/post1
set,last

esel,s,mat,,1
nsle,s,1
nsort,s,eqv,0,1
*get,yingli_max,sort,0,max
nsort,u,sum,0,1
*get,weiyi_max,sort,0,max
weiyi_max1=abs(weiyi_max)
allsel
*end

/input,zhamen_kkd,pds
/pds
pdanl,zhamen_kkd,pds

pdvar,tm,gaus,2.1e11,2.1e9,0,0
pdvar,bos,gaus,0.3,0.05,0,0
pdvar,midu,gaus,7850,50,0,0

pdvar,pp,gaus,1000*9.8*30,1000*9.8*30*0.04,0,0
pdvar,px,gaus,9810,50,0,0
pdvar,pd,gaus,1000*9.8*30*0.1*1.1,1000*9.8*30*0.1*1.1*0.135,0,0
pdvar,pn,log1,1000*9.8*30*0.2*1.2,1000*9.8*30*0.2*1.2*0.3,0,0
pdvar,yingli_max,resp
pdvar,weiyi_max1,resp

nn=10000
```

```
pdmeth,mcs,lhs
pdlhs,nn,1,mean
pdexe,pds_zhamen_kkd,,
```
